# Evaluation of a home-based parenting support programme—Parenting Young Children—For parents with intellectual and developmental disabilities when there is a risk for neglect: Study protocol for a multi-centre study

**DOI:** 10.1371/journal.pone.0318447

**Published:** 2025-02-10

**Authors:** Marie Holmefur, Tommie Forslund, Eva Randell, Birgitta Wennberg, Maria Ayoub, Lena Almqvist, Karin Fängström, Gunnel Janeslätt, Thomas Strandberg

**Affiliations:** 1 School of Health Sciences, Faculty of Medicine and Health, Örebro University, Örebro, Sweden; 2 Department of Social Work, Uppsala University, Uppsala, Sweden,; 3 Center for Social and Affective Neuroscience, Linköping University, Linköping, Sweden,; 4 School of Health and Welfare, Dalarna University, Falun, Sweden,; 5 School of Health, Care and Social Welfare, Mälardalen University, Västerås, Sweden,; 6 Department of Public Health and Caring Sciences, Uppsala University, Uppsala, Sweden,; 7 Center for Clinical Research Dalarna, Uppsala University, Uppsala, Sweden,; 8 School of Behavioural, Social and Legal Sciences, Örebro University, Örebro, Sweden; PLOS: Public Library of Science, UNITED KINGDOM OF GREAT BRITAIN AND NORTHERN IRELAND

## Abstract

**Introduction:**

Parents with intellectual and developmental disabilities (IDDs) often need parenting support, but there are few evidence-based programmes adapted to their cognitive needs. Parenting Young Children (PYC), a home-based programme for parents with IDDs, is perceived as beneficial by parents and practitioners, but it is unclear if PYC improves parenting. The purpose of the proposed mixed-methods study is therefore to evaluate the PYC programme for improved parenting in parents with IDDs.

**Methods and analysis:**

The quantitative evaluation will have a multi-centre, pretest-posttest study design and include parents with IDDs (children aged 0–9) in need of adapted parenting support. Goal-attainment in parenting skills, parental self-efficacy and child mental health will be measured outcomes. Interviews will be used to explore the perspectives of parents and children on PYC.

**Ethics and dissemination:**

Particpation is based on informed consent from parents and guardians of the participating children. Ethical approval was granted by the Swedish Ethical Review Authority.

## Introduction

Parents with intellectual and developmental disabilities (IDDs)—which comprise, e.g., intellectual disability (ID), Autism Spectrum Disorder (ASD) and Attention Deficit Hyperactivity Disorder (ADHD)—are overrepresented in social services [1–3]. In fact, these parents, who have cognitive difficulties in common, make up a notable proportion of all child protection cases [4,5]. Their children are also at markedly elevated risk for out-of-home placements [6–8], most commonly due to neglect and unsafe home environments [5,9,10]. In Sweden, parents are entitled to parenting support according to the Social Services Act (Chapter 3. § 6 a) [11]. Since 2008, a national strategy for parenting support prescribes the right to suitable support for all parents, including those with cognitive difficulties [12,13]. However, concerns have been voiced, in Sweden as well as internationally, about a lack of adapted, evidence-based interventions for parents with IDDs [4,5,14]. The home-based programme Parenting Young Children (PYC), initially developed for parents with ID [15], has shown promising results in qualitative studies [16,17]. PYC builds upon evidence-based cognitive support, and may therefore be suitable for a wider range of parents with IDD. Indeed, PYC is currently used broadly in Sweden for parents with IDD in perceived need of adapted parenting support. At the same time, knowledge gaps have been highlighted regarding PYC’s effectiveness and how children perceive the intervention [18]. Therefore, this protocol presents an evaluation of PYC for parents with IDDs who are at risk for child neglect.

Parents with IDDs such as ID, ASD and ADHD—undisputedly a heterogenous group, yet having cognitive challenges in common—are at risk for parenting problems [19,20]. Their children are also at risk for developmental delays and behavioural and mental health problems [21]. The parents are reporting lower levels of parental self-efficacy [22], a clinically relevant factor linked to both parent-child relationships, child development and parental well-being [23]. Parents with ID, who have limitations in intellectual and adaptive functioning [24], are at risk for difficulties in providing a safe home environment, and for insufficient skills for basic childcare and child-caregiver interactions [1,25]. They are also at risk of socio-economic adversity and limited social support. Parents with ID have elevated rates of child protection involvement [25–27]. Neglect due to insufficient care and unsafe home environments, and concerns regarding caregiver sensitivity, are common reasons for out-of-home placements [6,25]. While out-of-home placements are sometimes necessary to ensure the well-being of the child, they have significant consequences for both children and parents [27,28]. People with ASD and ADHD have limitations in executive functioning and self-regulating abilities [29] Although parenting in the context of ASD is understudied, parents with ASD report higher levels of caregiving difficulties [30,31] and lower parenting self-efficacy [32]. Moreover, parents with ASD report difficulties with demand-overload, child-caregiver play, adjusting to their children’s changing developmental needs, and overwhelming perceptual input [32,33]. Parents with ADHD are at risk for insufficient monitoring of and attention to their children and their needs, procrastination of parenting tasks, impulsive behaviours in relation to their child, less authoritative parenting, and difficulties in maintaining routines, planning, and ensuring predictability [19,34–36]. The problems could be even more pronounced in parents with higher levels of ADHD symptoms [34,37].

It is important to note that caregiving problems are multifactorially determined and do not solely pertain to the IDDs per se [38]. For instance, parents with IDDs often lack positive role models, have mental health problems, have experienced abuse and maltreatment, have limited social support, have low income, reside in impoverished neighbourhoods, and become parents early [4,39,40]. Parental IDD in and of itself is not an adequate justification for child removal, yet parents with IDDs sometimes suffer discrimination [41,42]. Parents with ID are, for example, over-reported to the social services, and their parenting is more closely scrutinised [26]. This may in part stem from negative assumptions about their ability to provide adequate care [5,30,43]. Negative attitudes and pessimism likely stem from lack of knowledge about these families and their service needs, and from insufficient access to adapted methods to support the families [10,26,44]. The social services thus face an important challenge in offering adapted support that meets the needs of these families [8]. Therefore, programs targeting these specific populations are needed.

### Support for parents with IDD to promote parenting

Parents with IDDs can acquire parenting skills if the support is adapted to their needs [3,14,45,46]. Reviewing research on interventions for parents with ID, Feldman concluded that interventions are most effective when they are skill-focused, situated in the home, use behavioural teaching strategies (e g, modelling, practice, feedback, and praise), and break down complex skills into smaller steps [47]. In line with these recommendations, programmes for parents with ID tend to be individualised and home-based [48,49], and to use learning materials and pedagogical techniques that demonstrate parenting skills in a form that the parents can understand [1]. Systematic reviews have also found promising results for both the parents and their children [3,46,50]. For instance, one study found that a home-based intervention improved parenting skills and the home environment, and thus reduced the risk for child removal [51]. Similarly, another intervention yielded lower levels of maladaptive parenting behaviours and child behaviour problems and increased parent and child well-being [45]. Improvements in parenting can also be maintained after program completion [49].

Support programmes for parents with ASD are scarce, and to our knowledge no such programme has been formally evaluated. For Parents with ADHD, the most commonly investigated intervention is pharmacological treatment, with limited effect on parenting ability [19,52]. Parental ADHD has also been found to diminish the effect of regular parenting programs, likely due to difficulties taking part in the programmes, and programmes not targeting the challenges experienced by the parents [52]. This indicates that support tailored to the challenges of parents with ADHD is warranted, especially when it comes to parents with high symptom levels [14]. One evolving programme tailored to parents with ADHD is the recently developed Improving Parenting Skills Adult ADHD (IPSA) programme [53]. Despite these promising findings, there are currently few evidence-based parenting support programmes adapted for parents with cognitive challenges. Considerably more evidence is also needed to understand the effects of parent training programmes for these parents and their children [3,46,54], and there is a need for better-quality research and long-term follow-up studies [55].

### Parenting Young Children

PYC is a home-based support programme originally developed for parents with ID who have children younger than seven years of age [15]. PYC aims to develop and improve parenting skills and strengthen parent-child interactions to reduce the risk of neglect [17]. PYC is based on Feldman and colleagues’ work on the Step-by-Step Parenting Program [56] and Parent-Child Interaction Therapy [57]. In line with Feldman’s recommendations, PYC is individualised and home-based, uses well-established social learning principles and behavioural teaching principles, breaks down complex skills into smaller steps, and emphasizes collaborating with the parents [15,47]. An initial evaluation with 24 Australian parents with ID found decreased parenting stress, increased parental self-efficacy, reduced child behaviour problems, and improved home-environments [15].

PYC was implemented in Sweden following a governmental initiative [17]. The programme was first translated and adapted through a collaboration between the developers and researchers in Sweden [16]. Subsequent empirical work then found that both parents and professionals perceived PYC as beneficial in developing parenting skills [15,17,58]. Professionals trained in PYC also found the programme suitable for a wider group of parents with cognitive difficulties as well as a wider age span of the children. Difficulties with executive functioning are for example common in different types of IDDs, and the strategies in PYC are directed at supporting executive functioning. Consequently, PYC is used in Sweden also for parents with marginal ID, ADHD, and ASD in need of adapted parenting support.

The Swedish Agency for Health Technology Assessment and Assessment of Social Services has highlighted a need for systematic evaluation of PYC’s effects on parents and children from different perspectives [18]. A quantitative evaluation with psychometrically sound measures warrants generalizable evidence and has the potential to form a solid base for evidence-based practice. Parents experiences of participating in PYC, and perceived changes in their parenting, must be evaluated since they are directly targeted by the programme. Children are rarely asked about their opinions of family interventions [59]. However, children are active producers of knowledge, and their experiences and perspectives are valuable [60]. Moreover, the United Nations Convention on the Rights of the Child underlines the importance of promoting children’s right to participate and to make their voice heard [61,62]. Children’s perspectives on their everyday situations must also be evaluated in the context of their natural environment, which for most children pertain to the family context [63]. While children’s experiences are of vital importance, there are to our knowledge no studies on children’s perspectives on daily activities and family situation after parents recieve PYC. This study is therefore motivated by the dual need to evaluate PYC using quantitative measures of improvement, and qualitative measures examining the perspectives of the parents and the children.

### Aims

The purpose of this study is to evaluate PYC for improved parenting in parents with IDDs when there is a risk for child neglect. Specifically, the following research questions will be addressed: 1) To what extent and how does PYC improve parental functioning in relation to goal attainment, parental self-efficacy, and child wellbeing? 2) How do parents describe their experiences of PYC? 3) How do children whose parents have received PYC describe their daily activities and interactions with parents?

## Methods and analysis

### Study design and study setting

The study will use a multi-centre, pretest-posttest design to evaluate PYC with quantitative measures. Further, a qualitative evaluation will involve individual interviews with parents who have received PYC and their children in order to explore their experiences. The study is registered in Clinicaltrials gov Reg no: NCT05935722. Registration was completed after recruitment of the first participant, because at the time of study start, prospective registration of studies was not common practice at the university then responsible for the study. The authors confirm that all ongoing and related trials for this intervention are registered.

The study will be conducted within the Swedish municipal social services. The municipalities will be purposively selected from different regions and will include small to large municipalities.

### Eligibility criteria

The study will include parents with IDDs (e g, ID, ADHD and ASD). Parents must have children aged 0-9 years at home and be assessed by social services as in need of adapted parenting support. If there are several children in the family, one child will be selected for the study evaluation. Exclusion criteria will be ongoing substance abuse, ongoing child abuse, and/or mental illness of such a nature and degree that it may interfere with parenting support. Further, children aged 4–9 years of parents who have undergone the PYC intervention will be recruited for interviews.

### Intervention-Parenting Young Children (PYC)

The PYC programme will be evaluated based on intervention periods typically lasting 6–12 months based on assessed needs. PYC is a home-based intervention programme involving weekly, one-hour sessions over the intervention period [15]. The programme has two core modules (1) Parent-Child Interaction and (2) Child Care Skills and Safety. The Parent-Child Interaction module focuses on the parent-child relationship and interaction skills such as responsiveness to the child’s signals, giving the child attention and encouragement, and supporting prosocial behaviour. The Child Care Skills and Safety module targets safety at home and the parent’s caring skills (e.g., food, health, and hygiene). Checklists are adapted to each parent according to his or her needs and are used to facilitate the PYC practitioner in helping the parent reach set goals for better parenting skills. The programme is compiled in a manual that includes work tasks, instructions for how to perform the tasks, and teaching materials.

There are two key components: PYC activities and PYC teaching strategies. PYC activities include outlining expectations, setting goals, developing an individually adapted intervention, teaching, and using checklists to track skill development. Teaching strategies used by the PYC practitioner include role play, coaching, and discrimination training.

To organise the learning of skills and to track parents’ development, the PYC-practitioner use task-analysis checklists, which break down complex caregiver behaviour into simple actions that can be trained in a stepwise manner. The checklists are initially used to assess the parent’s current level and then to guide step-wise teaching of a skill. The intervention also includes teaching strategies that facilitate implementation. Generalisation and maintenance of acquired skills and the significance of fidelity are emphasised, and different strategies and materials for how to achieve this are presented.

Professionals must attend a three-day training and follow-up training sessions to become PYC practitioners. Fidelity focuses on how well PYC activities and teaching strategies are implemented, and PYC practitioners are provided with checklists for self-evaluation [64].

### Outcomes

The study will use both quantitative and qualitative evaluations. The quantitative part of the study will be supplemented with a process evaluation.

### Quantitative evaluation

The primary outcome variable is goal-attainment in increasing parenting skills according to PYC’s intervention domains. This is motivated by PYC’s purpose of increasing perceived and real parenting ability, and by the structure of PYC, because parents continuously set goals for the intervention. All quantitative outcomes will be examined pre- and post-intervention, and 6 months post-intervention.

Goal-attainment in parenting skills: Goal-attainment in PYC’s intervention domains will be measured with the Canadian Occupational Performance Measure (COPM) [65,66]. COPM is an individualised, client-centred outcome measure that captures clients’ self-perception of everyday issues that influence their performance in daily life. COPM provides a basis for setting concrete individual intervention goals and for detecting changes in perceived performance and satisfaction with performance over time. The parents will select goals for the PYC-intervention and rate their performance and satisfaction using a 10-point rating scale. The performance scale ranges from 1 (“not able to perform it at all”) to 10 (“able to perform it extremely well”), and the satisfaction scale ranges from 1 (“not satisfied at all”) to 10 (“extremely satisfied”). The scale has been adapted with facial expressions illustrating each response option in order to facilitate responses. COPM assessments on parents’ performance will be gathered from both parents and PYC practitioners pre- and post-intervention. 6-month follow-up data will only be gathered from the parents, since cases may often be closed, in which case practitioners are not allowed to be in contact with the parents and won’t be able to report on their performance.

The COPM has demonstrated good validity, reliability, and sensitivity to change [67], with an increase of two or more points indicating a clinically significant change [68]. The clinical utility of COPM has also been demonstrated [67,69], and it has been used with individuals of different ages and diagnoses, including mental illness, ID, and ADHD [70–72].

Parental self-efficacy: The participants’ perception of their parenting self-efficacy will be assessed with the Parental Sense of Competence Scale (PSOC) [73]. The PSOC measures parents’ satisfaction with the parenting role, parenting efficacy, and interest in parenting. It has 15 items, rated on a 6-point scale ranging from 1 (”strongly agree”) to 6 (”strongly disagree”) [74]. PSOC has shown responsiveness to change and has been used with persons with ID, ADHD, ASD, and mental disorders [22,75,76].

Children’s mental health: Children’s mental health will be measured with parental ratings on the Strengths and Difficulties Questionnaire (SDQ) [77]. The SDQ is well-established for screening of mental health problems in 2–17-year-olds [78] and consists of 25 items divided into 5 subscales measuring emotional symptoms, conduct problems, hyperactivity/inattention, peer problems and prosocial behaviours [79]. The SDQ also includes an impact scale that measures overall difficulties, duration of difficulties, and whether difficulties cause burden, stress, or interfere with the child’s everyday life. The SDQ can be completed by parents and has demonstrated good psychometric properties [78–80]. It has been used successfully with parents with IDDs to evaluate parenting interventions and to investigate the social-emotional well-being of children of mothers with ID [45,81,82].

Demographic data: Three study-specific demographic forms will be collected pre-intervention: 1) parents will complete a form concerning their biological sex, date of birth, country of birth, living arrangement, family situation, education and work situation, cognitive difficulties (including possible diagnoses), and number of children in the household; 2) Social workers will complete a form concerning reasons for including a parent in the study (e g, possible diagnoses, observed cognitive difficulties) and previous contact and length of contact with social services; and 3) PYC-practitioners will complete a form about their age, biological sex, employing municipality, number of years in the social services, basic education, and education in PYC.

### Process evaluation

**Fig 1:**
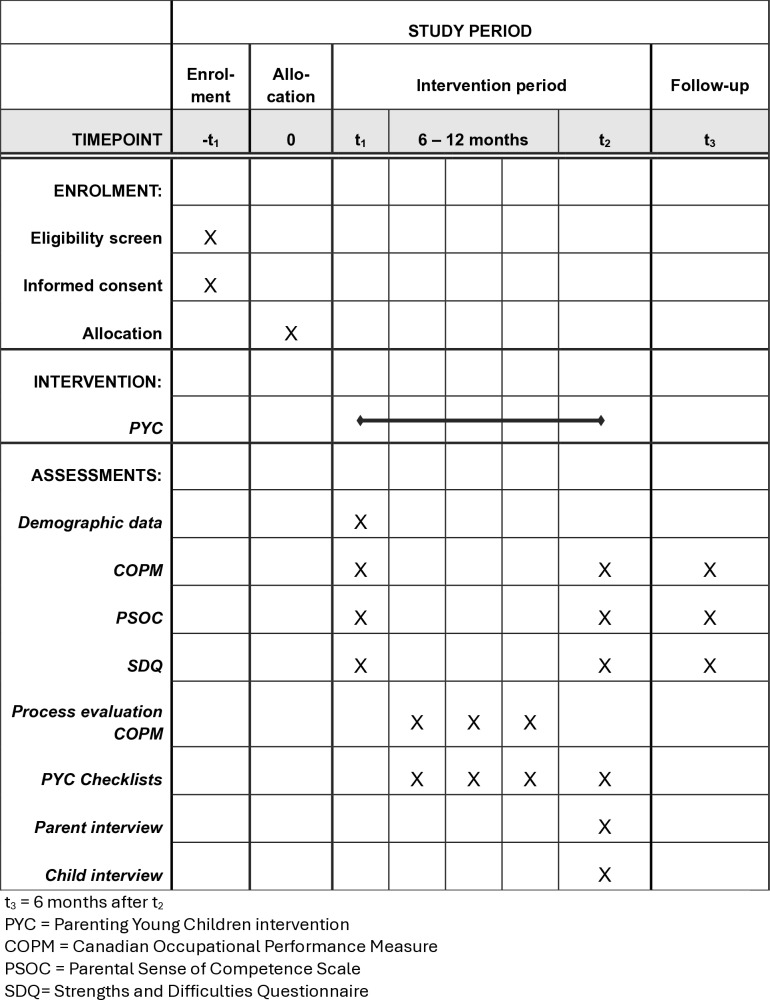
Participants’ flow through the study.

To evaluate the intervention process and to enable examination of response shifts [83], the parents will rate their performance and satisfaction using COPM once a month (Fig 1). Treatment fidelity will be monitored with PYC checklists, completed by the PYC practitioners, and with an intervention log for each participant documenting the date, the type of PYC activity, and the PYC teaching approach for each meeting.

### Qualitative evaluation

Qualitative methods will be used to explore the participants’ perspectives and perceptions.

Parents’ experiences: Parents’ experiences of PYC will be explored with semi-structured interviews. Parents will be interviewed individually, at a location of their choice, shortly after they complete PYC. A study-specific interview guide adapted for individuals with IDDs will be used [3]. The interview questions will include perceptions of parenting skills before and after PYC, positive and negative aspects of PYC, and PYC’s influence on parent-child relationships and other parts of their lives (e g, relationships).

Children’s perspectives: The children’s perspectives will be gathered through age-appropriate, semi-structured interviews, after their parents have received PYC. Children will be interviewed individually; at a place they and their parents have chosen. A study-specific interview guide has been designed, with age-appropriate wordings and time frames [84]. Children will be asked about their everyday activities and experiences, with different family members, in relation to different emotions. Both positive and negative emotions will be explored. Pictorial support will be used to facilitate children’s recollection and communication, influenced by methods using pictures and photos in interviews with children with and without disabilities [85–90]. To keep the children’s interest and attention, questions about what an ordinary day looks like are interspersed with tasks where the child can use pictures to tell the story [84]. First, pictorial support in the form of a circle will depict ordinary weekday and weekend days, respectively, with pictorial anchor points for waking up and morning routines(pre)school, extracurricular activities and family time, evening routines and going to bed. Additional pictorial support will be used, divided into pictures of morning and evening routines, important people, indoor and outdoor activities, and expressions of positive and negative emotions. The use of both visual and verbal parts helps children express their thoughts and possibly raise sensitive aspects of their family life [91].

### Participant timeline

Fig 1 describes the flow of participants through the study.

### Sample sizes

For the quantitative evaluation, a preliminary sample size calculation for one group and two-sided t-test with 80% power, to allow testing at *p* =  0.05 and an effect size of *d* =  0.5 rendered a sample size of 34 participants. When calculating on group standard deviation on COPM-performance of 2.04 and an expected mean increase of 1.47 from Clarke et al [92] the target sample size was adjusted to n =  18 participants. To account for potential attrition of up to 25%, the study will therefore include n =  24 participants. For the qualitative evaluations, 15 parents who have received PYC will be included and all children aged 4 years or older whose parent have received PYC will be asked to participate, with the aim to recruit 8-10 children.

### Recruitment

Participants will be identified and recruited by social workers at municipal social services that provide PYC. Before recruitment, social workers will participate in an introductory meeting about the project and about research ethics. Eligible participants will be enrolled continuously (consecutive sampling). Parents will be informed about the study using adapted verbal and written information (e.g., easy-to-read texts, supportive images). The researchers will work closely with and support the social workers in identifying and recruiting participants. Recruitment commenced on Sept 8^th^, 2022 and will be concluded on June 30^th^, 2025.

### Data collection methods

Data on goal-attainment in parenting skills (COPM) will be collected by the PYC-practitioners. A manual has been designed to reduce the risk of measurement error and each PYC-practitioner will partake in an instructional workshop on the COPM before starting data collection. The remaining data will be collected by the researchers or PYC-practitioners in interviews with the participants. The study-specific forms have been piloted for comprehensibility, and a manual has been developed to ensure standardised implementation.

### Retention

The PYC-practitioners will use a recruitment logbook to document participants who discontinue the intervention. To improve retention, the parents will receive a 100 SEK gift card at each data point (pre, post, follow-up, and interview), and the children will receive a book after the interview.

### Data management and confidentiality

Data will be managed and stored according to the General Data Protection Regulation in the EU and the data management policy at Örebro University. All participant information, interviews, and questionnaires will be stored in a password-protected storage system at Örebro University. Participants’ identity information will be replaced with code numbers, and the researchers will inform the PYC-practitioners about each participant’s code. A code list that links the participants’ identities to their unique codes will be established and stored separately from the research data in an encrypted file on a password-protected server. Only authorised staff will have access to these files. Data on paper forms collected by social workers will be stored securely at the social services facilities until they are transported to Örebro University. All researchers will have access to the data throughout the project.

### Patient and public involvement

A range of representatives for patients and the public has been and are involved in the design and conduct of this study. This includes representatives from patient organizations for people with ID, ADHD and ASD, the SUF Resource Center for parents with disabilities as well as the social service providers who assisted in collecting. These representatives were involved from the initial planning stage before funding was obtained. Since funding was secured, a reference group of representatives have continuously been consulted regarding design, practical implementation, burden of intervention and measures, and recruitment strategies. Further, they will be involved in the dissemination and implementation of study results.

### Statistical methods

Baseline characteristics will be reported by using summary statistics such as frequencies and proportions and mean/SD or median/IQR depending on the data distribution. The normality of outcome data will be assessed using the Shapiro–Wilks test. The primary comparison will be the change in goal-attainment (COPM) as rated by the parent and the PYC-practicioner, respectively, from baseline to post-intervention. Sensitivity analyses will be employed to evaluate the robustness of the results. Secondary analyses will involve changes in parenting self-efficacy (PSOC) and children’s mental health (SDQ) as well as follow-up measures for all outcomes. Effect sizes will be calculated as Cohens *d*. The process evaluation will involve repeated measures analyses of COPM data. Two-sided tests with a significance level of *p* <  0.05 will be employed. Missing data will be handled according to the recommendations of Jakobsen et al [93].

### Qualitative analyses

Content analysis and reflexive thematic analysis will be used for the parent and child interviews as appropriate [94,95,96]. The material will be coded by one researcher, and the codes will be reviewed by two co-authors. All co-authors of the resulting manuscript will participate in interpretation and discussion.

### Data monitoring

A formal data monitoring committee is not needed due to the minimal risks associated with participation. As such, stopping guidelines are not deemed necessary. An advisory group has been established, consisting of professional and NGO-organisations representing persons with IDDs. The research group will report to the advisory group and discuss any need for modifications or early termination of the data collection. An interim analysis will be performed when 50% of the data have been collected, and data collection will be stopped when the required number of participants is reached or when the principal investigator makes such a decision.

An adverse event is defined as any untoward occurrence believed to be causally related to participation in the study and that might result in inadequate caregiving or poor child safety. All adverse events occurring after study entry will be documented. The study will also examine the potentially negative effects of PYC through interviews with parents and children.

### Ancillary and post-study care

The parents’ ability to provide safety and meet their children’s needs may decline during the study. In such cases, necessary measures will be taken immediately in accordance with the Social Services Act. Parents will be interviewed by experienced researchers and social workers or similar. The interviewed children will be given the opportunity to talk about their experiences and feelings connected to their parent’s participation in PYC. The children will be met by a researcher with extensive experience in interviewing children who will treat them respectfully, and such conversations can serve as a tool to help the child process unpleasant experiences or events.

### Ethics approval and consent to participate

The study was approved by the Swedish Ethical Review Authority (Dnr: 2021-00771) on 11 March 2021. Social workers will inform eligible parents about the project and participation verbally and in writing. All information will be adapted to the parent’s intellectual level and will include information that participation is voluntary and can be withdrawn at any time without any negative consequences for the participants. Social workers will obtain written consent from eligible parents before inclusion. Guardians of eligible children will be informed verbally and in writing about the children’s interview. Before the interview start, written consent will be obtained from both guardians, and verbal consent will be sought from the children before commencing the interview. All methods will be performed in accordance with the relevant guidelines and regulations.

### Dissemination policy

The study results will be disseminated to stakeholders in society at a layperson level as well as through scientific communications (e.g., scientific journals and research conferences).

## Discussion

Parents with IDDs are at elevated risk for caregiving difficulties, and the lack of evidence-based support for this group is a concern for several reasons. For instance, the UN Convention on the Rights of Persons with Disabilities emphasises the right to parenthood and pertinent support. The Convention on the Rights of the Child became law in Sweden in 2020 and also emphasises the centrality of child-caregiver relationships, parenting support, and family preservation. Further, Sweden has adopted a national strategy with the goal of supporting all parents in their parenthood [12]. Research on parenting support for parents with IDDs is thus crucial in enabling society to fulfil its commitment to supporting families headed by parents with cognitive difficulties.

This study protocol presents a multi-centre study of the parenting support programme PYC. Qualitative studies of PYC have reported promising results with parents and PYC practitioners, indicating that the intervention may fill an important gap in social services. To date, several hundred Swedish social workers have been trained in PYC. However, the Swedish Agency for Health Technology Assessment and Assessment of Social Services has emphasised important knowledge gaps concerning the programme [18], including its effectiveness in improving caregiving and children’s mental health. The present study has been designed with these knowledge gaps in mind and can thus provide important knowledge. In addition to pre- and post-intervention assessments, the study will also include a 6-month follow-up to assess maintenance of effects and a process evaluation. Additionally, the study will add knowledge about PYC’s ability to improve parental self-efficacy and children’s mental health. Finally, the study will increase our understanding of parents’ perspectives of PYC, and of children’s perspectives on their family situations.

The choice of the COPM measurement instrument as outcome is based on the design of PYC where parents themselves set goals based on the needs that have emerged. In the COPM, the client rates both performance and satisfaction with performance, which can provide additional information about the experience of parenting. The PYC practitioner’s rating of the parent’s performance provides an additional dimension that can be seen as less subjective and which may reflect the social services’ view of the extent to which the parent has reached goal fulfillment. However, a potential bias to be aware of is that this can be perceived as an estimate of the PYC practitioner’s own effort toward the parent.

Evaluation of PYC within social services requires a design that is cognizant of professional and ethical values. A challenge that researchers often face when undertaking effectiveness studies in this context is the unwillingness to use random allocation [97]. Specifically, service providers often find random allocation to intervention and control groups unethical and in conflict with the professional values of providing adequate care for all. We encountered such concerns when preparing this study, with several municipalities that provide PYC finding it unethical to randomise parents with cognitive difficulties to treatment as usual, paired with an unwillingness of municipalities who do not practice PYC to participate at all. This resulted in the considerably weaker pretest-posttest design. Yet, there is currently only one quantitative study of PYC, and no Swedish study. The present study will consequently, nonetheless, contribute important knowledge.

Another challenge is associated with difficulties in asking social workers to be responsible for the recruitment of participants who are vulnerable and in need of their support. This is, however, the only way to identify and recruit participants. Through all steps, the voluntary nature of participation will be emphasised, and measures will be taken to adapt all information and the procedure to the parents’ and children’s cognitive levels. The researchers will also keep in close contact with the social workers by mail, phone, and visits in order to support the recruitment process and the data collection.

## Supporting Information

S1 File Supplemental file SPIRIT Checklist. (DOC)

S2 File Approved Ethics Study protocol English. (PDF)

## Abbreviations

ADHD: Attention Deficit/Hyperactivity Disorder
ASD: Autism Spectrum Disorder
COPM: Canadian Occupational Performance Measure
ID: Intellectual Disability
IDDs: Intellectual and Developmental Disabilities
PSOC: Parenting Sense of Competence Scale
PYC: Parenting Young Children
SDQ: Strengths and Difficulties Questionnaire.
