# Supplementary material for: Evaluation of a home-based parenting support programme—Parenting Young Children—For parents with intellectual and developmental disabilities when there is a risk for neglect: Study protocol for a multi-centre study
Source: PLoS One. 2025 Feb 10;20(2):e0318447. doi: 10.1371/journal.pone.0318447 (PMC11809872; doi:10.1371/journal.pone.0318447)
Supplement: S2 File — (PDF) [file pone.0318447.s002.pdf]

Research Plan for Ethics Application

Evaluation of Parenting Support Method – Parenting Young Children – in Home Environment for Parents with Cognitive Difficulties Where There is a Risk of Neglect.

Abstract

The research project evaluates Parenting Young Children (PYC), a home-based parenting support program tailored for parents with intellectual disabilities and other cognitive difficulties where there is a risk of neglect. The target group is vulnerable and found within the societal support system where living conditions may increase the risk of child placement if adequate support is not provided. According to the Social Services Act (SoL), the social services have the ultimate responsibility to ensure these parents receive the support they are legally entitled to, but there is a lack of evidence-based support programs adapted for this target group.

PYC, developed in Australia, has been implemented in many municipalities in Sweden since 2010, with good results according to clinical experiences and qualitative evaluations. However, there is a lack of evidence for PYC, which the Swedish Agency for Health Technology Assessment and Assessment of Social Services (SBU) has identified as an important knowledge gap. The purpose of this research project is therefore to fill this knowledge gap through a randomized controlled trial (RCT) study supplemented with process data and qualitative evaluation of the parents' and children's perspectives.

The research project is organized into three tracks and spans over five years. The project includes researchers with both clinical and research experience from the target group, and employs an interdisciplinary, mixed-method approach with researchers from various disciplines. The three tracks aim to study the following:

- Track I: Effect of intervention, based on an RCT design with multiple measurement points, in terms of increased parenting ability, parents' responsiveness to their children's signals, parents' self-efficacy, the child's mental health, and cost-effectiveness.
- Track II: Parents' experiences of PYC support studied qualitatively through interviews.
- Track III: Children's experiences of their family relationships and daily life before and after PYC, through interviews.

Keywords: Intellectual Disability, Cognitive Disability, Parenting Young Children, Parenting Support, RCT, Qualitative Method.

Background and Area Overview

Intellectual disabilities (ID) and other disabilities characterized by cognitive difficulties, such as attention deficit hyperactivity disorder (ADHD) and autism spectrum disorder (ASD), can be grouped under the umbrella term "intellectual and developmental disabilities" (IDD). While impairments in distinct aspects of cognitive functioning can be cross-diagnostic for different types of IDD, there are also characteristic differences. IDD is primarily characterized by limitations in intellectual functioning and adaptive behavior (e.g., Schalock et al., 2010), ADHD by difficulties with inattention and hyperactivity/impulsivity (Willcutt et al., 2012), and ASD by difficulties with social interaction and communication, as well as restricted behaviors and interests (Lord et al., 2018). At the same time, difficulties with executive functions have been observed in individuals with IDD (Bexkens et al., 2014), individuals with ADHD (Wilcutt et al., 2005), and individuals with ASD (Adams & Jarrold, 2012).

Parents with IDD are at increased risk for impaired parenting abilities, their children for developmental delays and behavioral problems, and the families for child placements in foster care (Starke, 2005). As many as 30–60 percent of all children of parents with IDD are placed in state care (Booth et al., 2006; Tøssebro et al., 2017) and it has been estimated that over 25 percent of all child welfare cases involve parents with IDD (Azar, Maggi, & Proctor, 2013; Booth & Booth, 2005; Tøssebro et al., 2017). The most common reason for child placement is neglect (Pecora et al., 2014), followed by concerns regarding responsiveness to children's signals (Alexius & Hollander, 2014). Parenting support interventions have proven to be a successful and cost-effective strategy for supporting parents and children (Socialstyrelsen, 2015), and Sweden has therefore adopted a national strategy for parenting support with the goal of supporting all parents. However, parents with IDD are not provided with the extent of support needed and there is a lack of evidence-based parenting support interventions adapted to their needs (Azar et al., 2013; Janeslätt et al., 2019a).

Parents with IDD and Parenting Ability

IDD has been established as a risk factor for inadequate parenting ability (Llewellyn et al., 2003b; Starke, 2005), which has been demonstrated using various research methods (Dowdney & Skuse, 1993; McConnell et al., 2011). Research using indirect methods has shown that parents with IDD have a measurable increased risk for custody investigations and placements of their children (e.g., Alexius & Hollander, 2014). Children of parents with IDD also have an increased risk of behavioral problems (e.g., Gillberg & Geijer-Karlsson, 1983; McGaw et al., 2007). As noted above, the most common reason for placements is neglect, including inadequate and unsafe home conditions, and insufficient care and supervision (Azar et al., 2012; Pecora et al., 2014). Parents with IDD may struggle to continuously provide for their children's care and meet their basic needs such as hygiene and nutrition. Children of parents with IDD are at

increased risk for mental health problems, accidents, violence, and abuse (Wickström, Höglund, Larsson & Lundgren, 2017).

Research on parenting behavior, for example through observations of parent-child interactions, has further found an increased risk of impaired parenting ability. It has been shown, among other things, that parents with IDD are at increased risk of interacting less with their children, being more directive, and less responsive to their children's signals (Crittenden & Bonvillian, 1984; Lindberg et al., 2017). A lower frequency of positive parenting behaviors, a higher frequency of negative parenting behaviors, and more inconsistent parenting behavior have also been observed among parents in the target group (Janeslätt & Roshanay, 2015). Responsiveness refers to parents' ability to perceive their children's signals, interpret them correctly, and respond to them adequately and quickly (Ainsworth et al., 1974). Responsiveness is an important aspect of parenting ability that has been associated with children's attachment quality (De Wolff & Ijzendoorn, 1997), brain development (Kok et al., 2015), and development of executive functions (Bernier, Carlson, Deschênes, & Matte-Gagné, 2012). Concerns about inadequate responsiveness among parents with IDD and their children's attachment quality are therefore common in custody cases involving parents with IDD (Alexius & Hollander, 2014). Further, a Swedish study found that mothers with mild ID had significantly lower levels of responsiveness to their children's signals compared to mothers without ID (Granqvist et al., 2014; Lindberg et al., 2017). Lower levels of responsiveness were also associated with an increased risk of disorganized attachment and externalizing behavior problems in their children. Among mothers with ID, experiences of abuse, trauma, and neglect in childhood were common and associated with lower levels of responsiveness and insecure attachment in their children. Overall, these results show that deficiencies in parenting ability can have a complex etiology that, in addition to cognitive difficulties, includes contextual risk factors.

Parenting ability consists partly of skills based on previous experiences with their own parents and others, as well as the individual's psychological state (George & Solomon, 2008). Consequently, impaired parenting ability also includes risk factors such as negative childhood experiences and experiences of abuse and trauma (Dilillo & Damashek, 2003; Stovall-McClough & Cloitre, 2006), psychological problems (Bakermans-Kranenburg et al., 2004), contextual factors such as poverty, and lack of social support (Cyr et al., 2010). Numerous studies have highlighted social vulnerability and unequal living conditions among individuals with IDD. Parents with ID often are single parents, live in poverty, reside in disadvantaged areas, are socially isolated, and are at high risk of developing mental health problems (Fernqvist, 2015; Ineland et al., 2009; Mineur et al., 2009). At the same time, individuals with IDD show a desire to become parents early, which may be partly due to the association of parenthood with becoming an adult (Janeslätt et al., 2019a; Rosqvist & Lövgren, 2013). A Swedish registry study found, for instance, that teenage parenthood is six times more common among

individuals with IDD (Höglund, Lindgren, & Larsson, 2012), which is a risk factor for both parents and children (Wahn & Nissen, 2008).

Overall, the causes of inadequate parenting ability in individuals with IDD appear to be complex. This necessitates the need for tailored support interventions aimed at parents with IDD. It is further emphasized that the interventions should focus on strengthening parents' ability to provide care and safety and interact positively with their children.

Parents with IDD, Child Placement, and Lack of Tailored Interventions

The increased risk that children of parents with IDD live under circumstances that may endanger the child's health and development is concerning. Sometimes, children need to be placed for protective reasons. However, it has also been highlighted that these parents are subject to more extensive scrutiny than other parents and that discrimination occurs (Alexius & Hollander, 2014; Booth et al., 2005). This can partly be due to stereotypical assumptions that cognitive difficulties inherently lead to an inability to provide adequate care (Llewellyn et al., 2003b). For example, a Swedish study found that a significant proportion of midwives believe it is inappropriate for women with mild ID to become parents (Höglund & Larsson, 2013). Such perceptions may stem from a lack of knowledge, as professionals have repeatedly expressed concern that the current lack of knowledge about these families makes it difficult to provide effective support (Bruno, 2012).

Placement in state care is a complex intervention with extensive consequences for both the children (Lindblad et al., 2013; Thomson & Thorpe, 2003) and the parents (Mayes & Llewellyn, 2009). Mothers with ID are often aware of the risk of their children being placed and can experience significant anxiety (Höglund & Larsson, 2013). In an interview study of mothers with IDD who had children placed in foster care, frustration and a need for tailored support to better understand placement decisions and to facilitate cooperation with social services and foster families were expressed (Janeslätt et al., 2019a). Additionally, foster care is not only a very costly intervention; research has found that foster placements can often be unstable solutions and pose a risk factor for the child's development (Brännström et al., 2017).

Sweden has adopted the United Nations Convention on the Rights of Persons with Disabilities (2008/09:28), which includes a right to parenthood. This includes, among other things, providing appropriate support to all parents. According to Article 2 of the Convention on the Rights of the Child, all children have the right to good living conditions. This entails ensuring that all children have equal opportunities to benefit from various forms of parenting support aimed at parents. The Social Services Act also states that parents have the right to receive the support they need in their role as parents (SoL 3, Chapter 6 a). Thus, it is a societal responsibility to support these families. However, parents with IDD do not receive the extent of support they need, and the

support available is not sufficiently tailored to the target group (Azar et al., 2013; Charlton et al., 1998; Janeslätt et al., 2019b; Mayes et al., 2011).

Studies show, for example, that there is moderately strong evidence that cognitive support is effective in daily routines for people with cognitive difficulties (Arvidsson et al., 2013; Gillespie et al., 2012). Examples of such support include visual aids and support for organizational skills, i.e., support in planning how to perform various tasks, with concrete step-by-step instructions (micro prompting and task sequencing). Furthermore, several studies have shown that methods aimed at parents with IDD, which structurally adapt the support to the target group, are effective (Azar, Maggi, & Proctor, 2013; Feldman, 1994; Feldman, Ducharme, & Case, 1999; Wade, Llewellyn, & Matthews, 2008).

Parents with IDD, Parenting Young Children (PYC), and Knowledge Gaps

A parenting support method that uses existing knowledge about cognitive support is Parenting Young Children (PYC), created by Catherine Wade and Robyn Mildon in Australia for parents with IDD who have children aged 0-6 living at home (Starke, Wade, Feldman, & Mildon, 2013). PYC is conducted in the parents' home together with a trained PYC facilitator, with support and information tailored to the parent's individual needs. The method focuses on two areas: care and interaction. PYC includes about 60 checklists for recurring everyday situations in these two areas that can be adapted and further developed in collaboration between the PYC facilitator and the parent. After clarifying the roles of the parent and the PYC facilitator, the parent chooses a goal to work towards and then works on that goal together with the PYC facilitator until it is achieved. In connection with the parent setting a goal, observations are also conducted in the home and the intervention is developed for the individual and their wishes, often with the help of visual aids. The parent then evaluates the intervention and perceived development in relation to the goal, and either a new goal is set for continued intervention, or the intervention with PYC is concluded. PYC has no standardized intervention time but usually takes 6 to 12 months.

The parent-centered work with own goals responds to ethical requirements regarding participation and facilitates both parents' motivation and cooperation between the parent and the PYC facilitator. By focusing on one goal at a time and working practically in the parents' home, PYC is also adapted to these parents' cognitive abilities, which can facilitate long-term learning and the ability to generalize skills. PYC and its approach have been perceived as suitable not only for parents with IDD but also for other parents who may need home-based intervention based on knowledge about cognitive support. Therefore, PYC is used in Sweden today for parents with cognitive difficulties.

PYC has been translated into Swedish (McHugh, Wade, Mildon, & Starke, 2014) and has been qualitatively evaluated with promising results in Australia and Sweden (Elaine Mc Hugh & Starke, 2015; Starke et al., 2013), as well as in Norway (Thronsen et al., 2014).

PYC has been tested, developed, and adapted to Swedish conditions in various stages. Initially, as a project from 2011-2014 in close collaboration between the "Parenting Research Centre in Melbourne", the Department of Social Work at the University of Gothenburg, FoU Sjuhärad Welfare, the Gothenburg Region FoU in the West, FoU Center for Care, Welfare, and Social Work Linköping, and FoU Support Region Federation Uppsala (Mensas, 2014). This work was conducted partly with funds from the Swedish Inheritance Fund. Research from the University of Gothenburg was conducted with grants from Forte (Elaine Mc Hugh & Starke, 2015; McHugh et al., 2014; Starke et al., 2013). A recently published feasibility study showed that the implementation of PYC was feasible, with generally positive attitudes towards evidence-based practice, good program compatibility, low perceived program complexity, and high satisfaction with implementation support. However, there were indications of problems in how the program was conducted by some PYC facilitators: parents received fewer than the recommended number of sessions, and observation of parents and follow-up were rarely used by the PYC facilitators (E Mc Hugh & Starke, 2020).

Since 2015, the Swedish development and training work of PYC has been managed by a consortium where FoU Sjuhärad Welfare, the Gothenburg Region FoU in the West, FoU Center for Care, Welfare, and Social Work Linköping, and SUF Knowledge Center, Close Care and Health, Region Uppsala. The consortium is non-profit and financed by training revenues. The aim of the consortium is for the parties to take joint responsibility for managing, developing, and ensuring the quality of the implementation of the PYC training program in Sweden and Scandinavia and to provide a sustainable home for the dissemination of PYC in the Nordic countries. During the project period, about 240 people were trained in PYC. Today, PYC has been implemented in many Swedish municipalities and over 400 people have now been trained as PYC facilitators. Still, there is no quantitative evaluation of effectiveness, a knowledge gap that has recently been highlighted as a priority research area in the development of a new national strategy to strengthen parenting support (SBU, 2018).

SBU (2018) calls for research evaluating whether PYC can improve parents' caregiving and reduce the risk of neglect, research with validated outcome measures for both parents and children, and research investigating any negative effects of PYC. SBU (2018) advocates for multicenter studies, evaluation of health economic aspects, a combination of quantitative and qualitative methods, and longitudinal follow-ups. This project is designed to address the knowledge gaps identified by SBU and therefore includes both quantitative methods to evaluate the effectiveness of PYC and qualitative methods to collect both parents' experiences of PYC and children's perspectives.

Does PYC Improve Care and Interaction and Reduce the Risk of Neglect?

PYC aims to achieve improvements in the goals that parents identify within PYC's areas of interaction and care; strengthen parents' ability to meet their children's needs, take

care of their children, provide a safe environment, and interact (responsively) with their children. Since concrete goals are an important tool in the work with PYC, the method should be evaluated in terms of goal achievement. This also offers an evidence-based way to assess the extent to which parents reach the goals they have identified themselves (Law et al., 2005).

In addition to goal achievement, PYC should also be evaluated in terms of its ability to improve parents' sense of self-efficacy and their responsiveness. Responsiveness is a component that, to varying degrees, has been integrated into most parenting programs offered in Sweden today. If PYC, through concrete and tailored support, can offer positive experiences, it can strengthen parenting ability. This, in turn, can be assumed to result in a sense of empowerment in parenting ability, which is expected to increase parents' sense of self-efficacy. This self-efficacy can also be reliably measured with well-evaluated questionnaires (Malmberg et al., 2003). Responsiveness, on the other hand, is usually measured through semi-structured observations of parent-child interactions that are video-recorded and later coded by coders who are blind to the treatment conditions. Measuring responsiveness, therefore, offers an "objective" measure of the extent to which participation in PYC improves a distinct aspect of parents' ability to interact with their children.

The need to evaluate whether PYC can generate improvements in care and interaction is related to the parent's role in the child's development and the fact that supporting the child's development is a goal for parenting programs. It is therefore important to also examine whether PYC produces effects that can be generalized to the child's mental health, such as increased functioning at home and in other contexts such as with peers and in preschool. Children's functioning can be reliably assessed by their parents, who know them well and have seen them in many different situations (Rydell, Berlin, & Bohlin, 2003).

SBU highlights the need for a health economic evaluation where the costs of providing PYC are compared with the costs of treatment as usual (TAU) in relation to potential gains in the parent's quality of life and the child's mental health. The health economic evaluation includes the costs of care for children and parents, both the need for emergency care, health care interventions such as child health center visits, and other care. A desirable consequence of parenting support is that the child does not need to seek emergency care due to accidents or violence, but that the parent actively seeks support from health care for various health conditions that affect children in general as well as the children in the study.

How is PYC Experienced by Parents and Children?

Interviewing parents with IDD should provide a better understanding of the PYC program, whether it is perceived as beneficial, and if it has any negative effects. There is a need to understand parents' expectations and experiences of participation and to

investigate whether parents feel that participation in PYC has affected their parenting ability. There is also a further need to understand whether participation affects other parts of life and to highlight social aspects related to experiences of PYC, such as how the parent experiences the support of the PYC facilitator.

Children are rarely asked about their own experiences of the support the family receives and whether it leads to any positive or negative changes from their perspective. The child's perspective on everyday life situations must be evaluated in the child's natural environment, which for most children includes family life (McConachie, Colver, Forsyth, Jarvis, & Parkinson, 2006; Sommer, Samuelsson, & Hundeide, 2009). To truly understand children's everyday lives, the researcher must directly address the children (Ben-Arieh, 2012). Regarding research on preschool children's perspectives, there are no such studies at all. One way to involve children in the evaluation of parenting interventions is by conducting interviews with the children of the parents who participate. In fact, even very young children can reliably describe their experiences (Hershkowitz, Lamb, Orbach, Katz, & Horowitz, 2012), although the interview method must be adapted to the children's cognitive level to be acceptable, facilitate communication, reduce power imbalances between the adult interviewer and the child, and alleviate potential stress associated with the interview situation.

Does PYC Improve Care and Interaction and Reduce the Risk of Neglect?

PYC aims to achieve improvements in the goals that parents identify within PYC's areas of interaction and care; strengthen parents' ability to meet their children's needs, take care of their children, provide a safe environment, and interact (responsively) with their children. Since concrete goals are an important tool in the work with PYC, the method should be evaluated in terms of goal achievement. This also offers an evidence-based way to assess the extent to which parents reach the goals they have identified themselves (Law et al., 2005).

In addition to goal achievement, PYC should also be evaluated in terms of its ability to improve parents' sense of self-efficacy and their responsiveness. Responsiveness is a component that, to varying degrees, has been integrated into most parenting programs offered in Sweden today. If PYC, through concrete and tailored support, can offer positive experiences, it can strengthen parenting ability. This, in turn, can be assumed to result in a sense of empowerment in parenting ability, which is expected to increase parents' sense of self-efficacy. This self-efficacy can also be reliably measured with well-evaluated questionnaires (Malmberg et al., 2003). Responsiveness, on the other hand, is usually measured through semi-structured observations of parent-child interactions that are video-recorded and later coded by coders who are blind to the treatment conditions. Measuring responsiveness, therefore, offers an "objective" measure of the extent to which participation in PYC improves a distinct aspect of parents' ability to interact with their children.

The need to evaluate whether PYC can generate improvements in care and interaction is related to the parent's role in the child's development and the fact that supporting the child's development is a goal for parenting programs. It is therefore important to also examine whether PYC produces effects that can be generalized to the child's mental health, such as increased functioning at home and in other contexts such as with peers and in preschool. Children's functioning can be reliably assessed by their parents, who know them well and have seen them in many different situations (Rydell, Berlin, & Bohlin, 2003).

SBU highlights the need for a health economic evaluation where the costs of providing PYC are compared with the costs of treatment as usual (TAU) in relation to potential gains in the parent's quality of life and the child's mental health. The health economic evaluation includes the costs of care for children and parents, both the need for emergency care, health care interventions such as child health center visits, and other care. A desirable consequence of parenting support is that the child does not need to seek emergency care due to accidents or violence, but that the parent actively seeks support from health care for various health conditions that affect children in general as well as the children in the study.

How is PYC Experienced by Parents and Children?

Interviewing parents with IDD should provide a better understanding of the PYC program, whether it is perceived as beneficial, and if it has any negative effects. There is a need to understand parents' expectations and experiences of participation and to investigate whether parents feel that participation in PYC has affected their parenting ability. There is also a further need to understand whether participation affects other parts of life and to highlight social aspects related to experiences of PYC, such as how the parent experiences the support of the PYC facilitator.

Children are rarely asked about their own experiences of the support the family receives and whether it leads to any positive or negative changes from their perspective. The child's perspective on everyday life situations must be evaluated in the child's natural environment, which for most children includes family life (McConachie, Colver, Forsyth, Jarvis, & Parkinson, 2006; Sommer, Samuelsson, & Hundeide, 2009). To truly understand children's everyday lives, the researcher must directly address the children (Ben-Arieh, 2012). Regarding research on preschool children's perspectives, there are no such studies at all. One way to involve children in the evaluation of parenting interventions is by conducting interviews with the children of the parents who participate. In fact, even very young children can reliably describe their experiences (Hershkowitz, Lamb, Orbach, Katz, & Horowitz, 2012), although the interview method must be adapted to the children's cognitive level to be acceptable, facilitate communication, reduce power imbalances between the adult interviewer and the child, and alleviate potential stress associated with the interview situation.

Interviews with children can highlight several important issues. For example, children may have difficulty understanding decisions made and the reasons behind them, or they may feel that a parenting support program has negative effects. Additionally, children may attribute the family's difficulties to proximal factors such as the parents' inability to support the child or to more distal contextual factors such as financial difficulty and lack of social support. Environmental risk factors can also interact with risk factors related to the children, such as developmental delays or behavioral problems.

In summary, the research project will seek to answer several of the knowledge gaps identified by SBU (SBU, 2018). The project includes an assessment of PYC's ability to improve care and interaction with well-validated instruments and collects experiences from both parents and children. Additionally, the project combines quantitative and qualitative methods in a mixed-method approach.

Purpose

The overall aim of the project is to evaluate the effectiveness of PYC through a randomized controlled trial (RCT) with quantitative data as well as qualitative data in the form of parent and child interviews. This is to promote scientific understanding of:

1. PYC's effectiveness compared to usual treatment (treatment as usual; TAU) in terms of improving: a. Parenting ability, measured by goal achievement within PYC's modules (i.e., care/safety, interaction), b. Parents' responsiveness to children's signals, measured through semi-structured observations of interaction situations, c. Self-rated parenting competence (i.e., parents' confidence in their parenting role), measured by self-rated parenting competence, d. Child's mental health, measured by mental health assessments, e. Cost-effectiveness.
2. Parents' experiences of participating in PYC, collected through qualitative interviews.
3. Children's experiences of their family relationships and family everyday life before and after the implementation with PYC, collected through qualitative interviews.

Method and Design

The project will use a randomized controlled design to compare PYC with clinical routine (treatment as usual, TAU), in close collaboration with PYC facilitators. Measurements in the RCT are made before, after, and 12 months after the intervention. The quantitative RCT will be supplemented with qualitative methods to collect experiences from parents and children.

Target Group: Parents with cognitive impairments who have children aged 0-6 living at home and need tailored support in parenting competence (in the form of PYC) at home. Children (3-6 years) of participating parents will also be asked to participate.

Exclusion Criteria: Parents with active substance abuse and/or with mental illness of such nature and degree that it may affect participation.

Intervention

PYC will be compared with TAU, the interventions and support usually offered to parents in similar situations.

Intervention with PYC

PYC is a home-based parenting support program for professional social workers who meet parents with cognitive difficulties who have children younger than seven years living at home. PYC is based on well-proven social pedagogical principles integrated with knowledge of evidence-based cognitive support. To use the program, professionals need training to become PYC facilitators and receive methodological support. The basic training includes three days, and methodological support is then provided in groups at seven sessions about once a month. There is also additional training comprising two days with continued methodological support. More information about the program is available on the website www.pyc.se.

The program's process is summarized in a manual with four sections:

Section 1: Developing the Intervention Here, the respective tasks and roles of the parent and the PYC facilitator during the training are clarified.

Section 2: Teaching Strategies This section is aimed at the PYC facilitator and addresses general principles of teaching, various teaching strategies, pedagogical methods, and help and tips in teaching parents.

Section 3: Care and Interaction This section deals with the care and upkeep of the child as well as training interaction skills.

Section 4: Approaches for Successful Implementation This section covers various educational techniques that increase the likelihood of successful program implementation.

The PYC manual emphasizes the importance of developing a mutual understanding of the collaboration between parent and PYC facilitator, building trust, and clarifying the respective tasks and roles of parents and PYC facilitators, which is explained during training.

The intervention is divided into two modules:

Module 1 – Care This module addresses the care and upkeep of the child as well as safety aspects. The purpose of the care module is to provide the parent with practical knowledge about the child's basic needs and develop skills to meet these needs. Checklists for various situations, adapted to different ages, are available to assist both the PYC facilitator and the parent.

Module 2 – Interaction This module addresses training interaction skills. The parent is trained in relationship-building situations aimed at increasing responsiveness to the child and providing the child with stimulation and attention. Additionally, the module includes training the parent's communication with the child to promote the child's language development. Checklists for various situations related to the two modules, adapted to children's different ages, are available to assist facilitators and parents.

Intervention with Treatment As Usual (TAU) Parents with intellectual disabilities (ID) or other cognitive difficulties need support in their parenting role, and it is the responsibility of social services to ensure that children and families receive the advice and support prescribed by the Social Services Act (SoL). However, there are various forms of support, and the support may vary between municipalities. The most common forms of support for parents with IDD and their children are individually oriented interventions and guidance provided to the parents, contact person or contact family for the children, and placement in foster care. Family therapists who regularly visit families and work in their homes can support the parents; contact persons for the children can do activities with the children based on their needs and wishes; and contact families can take the child into their home, including overnight stays. Social services also offer group activities that can be directed at parents or children. The project will therefore document and classify the support given to all participants receiving TAU, according to the National Board of Health and Welfare's guidelines for classification of its activities. This allows for a comparison of PYC support and TAU. Parents randomized to the control group will receive TAU for 6-12 months and will be offered PYC intervention if needed after the 12-month follow-up after the TAU intervention is completed. In cases where parents are deemed to need PYC earlier, PYC will be offered as soon as needed after the post-measurement after TAU is completed.

Track I: Quantitative Evaluation of PYC's Effect Compared to TAU Two primary outcome measures will be used: goal achievement and parents' responsiveness. These, along with a number of secondary outcome measures, will be examined before and after the intervention and 12 months after the intervention.

Goal Achievement in Parenting Ability The outcome measure, goal achievement in PYC's intervention modules, will be measured with the Canadian Occupational Performance Measure (COPM; Law et al., 2005). COPM is a standardized, client-centered outcome measure that enables individuals to identify, validate, and prioritize everyday problems that limit or affect their performance in daily activities. COPM provides a basis for setting individual goals for the intervention and for detecting changes in the person's own perception of performance and satisfaction with performance over time. In this study, parents with IDD will choose goals from the PYC modules and rate their own performance and satisfaction with an adapted VAS scale. The performance scale ranges from "cannot perform at all" to "can perform extremely well," and the satisfaction scale ranges from "not satisfied at all" to "extremely satisfied." COPM has shown good reliability, validity, and sensitivity to change (Carswell et al., 2004; Sakzewski, Boyd, & Ziviani, 2007); an increase of two or more points indicates a clinically significant change (Law et al., 2005). The clinical usability of COPM as an outcome measure has also been demonstrated (Carswell et al., 2004; Wressle,

Eeg-Olofsson, Marcusson, & Henriksson, 2002), and it has been used with people of various ages and diagnoses, including mental illness and IDD (Enemark Larsen, Rasmussen, & Christensen, 2018; Harr, Dunn, & Price, 2011; McColl et al., 2005; Sakzewski et al., 2007).

Parents' and PYC facilitators' ratings of performance and satisfaction (COPM) will be collected once a month. This is to enable process evaluation and because it is common for people with disabilities to rate their performance highly even at the first measurement (t1) since they are not fully aware of what should be included in the activity to be rated, COPM ratings will be collected at multiple points. Additionally, the checklists included in the PYC intervention, which are continuously filled in by PYC facilitators, will be used as process measures.

Observations of Parental Responsiveness The outcome measure, parental responsiveness, will be measured before and after the intervention in 20-minute long, semi-structured observations of parent-child interaction. We will use various subsections designed to subject parents to different challenges that mimic everyday demands to increase the validity of the observations. For example, parents will not only play with their child, with and without toys, they must also complete a task while remaining responsive to their children. This procedure is in line with recommendations in international research and follows our previous study (Lindberg et al., 2017). The observer will bring standardized age-appropriate toys, and the interaction situations will be video recorded. The observations will be blindly coded for group affiliation (PYC, TAU) and measurement occasion (pre, post) using Ainsworth's responsiveness scales (Ainsworth, 1974; cf. Granqvist et al., 2014). These scales, which provide continuous scores (1-9), are not only widely used and well validated, but have also been used effectively in previous research on responsiveness interventions (van IJzendoorn, Juffer, & Duyvesteyn, 1996).

Self-Rated Parenting Ability Participants' perception of their parenting skills will be evaluated with the Parental Sense of Competence Scale (PSOC). PSOC is a questionnaire with 15 statements answered on a 6-point scale from 1 (strongly agree) to 6 (strongly disagree) (Roger & Matthews, 2004). PSOC has been psychometrically evaluated and has been shown to measure satisfaction, efficacy, and interest in the parenting role (Roger & Matthews, 2004; Gilmore & Cuskelly, 2009). When used as an evaluation measure, PSOC has shown sensitivity to change (Lange et al., 2018; Reedtz et al., 2011) and has been used with mothers with ADHD (Sonuga-Barke et al., 2002) and individuals with mental, neuropsychiatric, and intellectual disabilities (Roshanay et al., 2019).

Observations of Parental Responsiveness The outcome measure, parental responsiveness, will be measured before and after the intervention in 20-minute long, semi-structured observations of parent-child interaction. We will use various subsections designed to subject parents to different challenges that mimic everyday demands to increase the validity of the observations. For example, parents will not only play with their child, with and without toys, they must also complete a task while remaining responsive to their children. This procedure is in line with recommendations

in international research and follows our previous study (Lindberg et al., 2017). The observer will bring standardized age-appropriate toys, and the interaction situations will be video recorded. The observations will be blindly coded for group affiliation (PYC, TAU) and measurement occasion (pre, post) using Ainsworth's responsiveness scales (Ainsworth, 1974; cf. Granqvist et al., 2014). These scales, which provide continuous scores (1-9), are not only widely used and well validated, but have also been used effectively in previous research on responsiveness interventions (van IJzendoorn, Juffer, & Duyvesteyn, 1996).

Self-Rated Parenting Ability Participants' perception of their parenting skills will be evaluated with the Parental Sense of Competence Scale (PSOC). PSOC is a questionnaire with 15 statements answered on a 6-point scale from 1 (strongly agree) to 6 (strongly disagree) (Roger & Matthews, 2004). PSOC has been psychometrically evaluated and has been shown to measure satisfaction, efficacy, and interest in the parenting role (Roger & Matthews, 2004; Gilmore & Cuskelly, 2009). When used as an evaluation measure, PSOC has shown sensitivity to change (Lange et al., 2018; Reedtz et al., 2011) and has been used with mothers with ADHD (Sonuga-Barke et al., 2002) and individuals with mental, neuropsychiatric, and intellectual disabilities (Roshanay et al., 2019).

Children's Mental Health The children's mental health will be measured with the Strengths and Difficulties Questionnaire (SDQ; Goodman, 1997). SDQ is an internationally well-established instrument used for screening mental health problems in 2-17-year-olds, focusing on behavioral and emotional symptoms and peer relationships (Smedje, Broman, Hetta, & von Knorring, 1999). The questionnaire can be completed by multiple informants, including parents and teachers, and has demonstrated good psychometric properties (Dahlberg, Ghaderi, Sarkadi, & Salari, 2019; Goodman, 2001; Smedje et al., 1999; Stone et al., 2015). The instrument has also been used in previous research to evaluate interventions for parents with IDD (Glazemakers & Deboutte, 2013) and to investigate social-emotional well-being in children of mothers with IDD (Hindmarsh, Llewellyn, & Emerson, 2017). In these studies, parents completed the questionnaire themselves, indicating that SDQ can be used by parents with IDD. The SDQ consists of 25 questions that can be divided into 5 subscales measuring emotional symptoms, behavioral problems, hyperactivity/inattention, peer relationship problems, and prosocial behavior (Goodman, 2001). Swedish norms are available for preschool children rated by parents and preschool teachers (Dahlberg, Fält, Ghaderi, Sarkadi, & Salari, 2019).

Cost-Effectiveness of the Intervention As a health economic evaluation, a health-cost analysis will be conducted by collecting and comparing costs and quality of life for PYC and TAU. Parents' quality of life will be measured using the quality of life instrument Assessment of Quality of Life 6D (AQoL-6D, Richardson et al., 2012). AQoL 6D has 20 questions in the dimensions of independent living, relationships, mental health, coping, pain, and senses, which are modeled separately and then combined into a single score. Additionally, the SDQ and data on healthcare utilization collected with a study-specific form will be used as the basis for the health economic evaluation.

Data will be collected in individual meetings with the parent. If needed, the parent will be offered help to read or explain the questions in the self-assessment forms (PSOC, SDQ, AQoL 6D).

Track 2 – Qualitative Evaluation of Parents' Experiences with PYC

Parents participating in the study will be interviewed shortly after completing the PYC program (n = 15). A study-specific interview guide will be used with questions adapted for individuals with IDD (Azar et al., 2013). The interview guide is divided into four selected areas with open questions. If necessary, alternative, more directed questions will be used. The interview guide will be piloted.

Key areas include: a. Parents' perception of parenting ability before PYC. b. Parents' perceptions of how PYC has been adapted to and suited the family's need for support. c. Parents' perception of parenting ability after PYC and the impact of PYC on other aspects of life. d. Unexpected positive or potential negative effects of PYC.

Interviews will be conducted in the parent's home or at a location of their choice. The interviews will be analyzed using qualitative content analysis, a method suitable for exploring similarities and differences in the data material (Elo & Kyngäs, 2008; Graneheim & Lundman, 2004). The interviews will be conducted by researchers experienced in interviewing individuals with IDD.

Track 3 – Qualitative Evaluation of Children's Perspectives

Two types of interviews will be conducted before and after the PYC intervention to capture the children's perspectives. Children aged 3-6 years will be interviewed using "In My Shoes" (IMS; n = 20), and children aged 6 years will participate in "Photo Elicitation Interview" (PEI, n = 12). Six-year-old children will be offered one of the two interview types. The two interview techniques capture different aspects of children's experiences: IMS focuses on the emotional and relational aspects of family experiences, while PEI focuses on daily life activities. There are no exclusion criteria except for age. Both IMS and PEI interviews will be transcribed and analyzed using qualitative content analysis since the need is not for an in-depth understanding of a phenomenon but a description of experiences produced by the respondents (Graneheim & Lundman, 2004).

IMS is an interactive, computer-based interview tool designed to help children verbalize their experiences, thoughts, and feelings in various environments and in relation to different people (Calam, Jimmieson, Cox, Glasgow, & Groth Larsen, 2000; Fängström, 2017; Grasso, Atkinson & Jimmieson, 2013). IMS is suitable for children from 3 years old and is enjoyed by children. It consists of a series of modules with stylized icons of places, people, feelings, speech, thoughts, and emotions. The icons serve as visual aids for children's communication as they can help retrieve memory images and provide children with multiple ways to express themselves. For example, they can point at the screen, click with the mouse, or write. A trained interviewer sits side by side with the child and uses IMS together with the child, making it a triadic interview (Calam, Cox, Glasgow, Jimmieson, & Groth Larsen, 2000). The goal is to open areas for

communication and enable the child to express themselves freely (Bøhren & Stabrun, 2013). IMS is believed to reduce the power imbalance between the adult interviewer and the child and alleviate potential stress associated with the interview situation.

IMS has been validated and found to be a reliable method (Fängström et al., 2016). It has also been found useful with children of various preschool ages to help them express their subjective experiences with both detail and depth (Bokström, Fängström, Calam, Lucas, & Sarkadi, 2016), and it helps shy and less communicative children to communicate (Fängström, Salari, Eriksson, & Sarkadi, 2017). IMS is primarily used within the framework of social services in various places in Sweden and as part of various research projects where children's perspectives are sought. For example, in an ongoing study at Uppsala University, In My Shoes is used to evaluate the parenting program Triple P from the children's perspective.

Interviews will be filmed and conducted in conjunction with the parent starting and completing the PYC intervention. IMS interviews are semi-structured, and the children will be asked about their experiences in the family related to various emotions and family members. Both positive and negative feelings will be explored. In accordance with recommendations for interviews with young children, we primarily ask open-ended questions and more directed questions if necessary. The interviews will be conducted by researchers trained in In My Shoes and experienced in conducting interviews with young children.

Photo Elicitation Interview (PEI) is an innovative method based on visual data research and involves photography and subsequent interviews with the child (Almqvist & Almqvist, 2015; Einarsdottir, 2005; Epstein, Stevens, McKeever, & Baruchel, 2006; Ford et al., 2017; Hong & Goh, 2019; McCloy, White, Lee Bunting, & Forwell, 2016). Children are asked to take photographs of meaningful daily life situations as a way to access their perceptions and experiences. These photographs are then used in individual interviews with the children to facilitate the children's descriptions of their perceptions and experiences of meaningful daily life situations.

Children will be offered to borrow a digital camera, but they can also use their own camera if it is more familiar to them. The children will be encouraged to take as many photos as they want of their daily situations in their immediate environment over a period of two weeks. If children want to take pictures in contexts such as their preschool or leisure activities, permission must be obtained. The children's parents will be asked to support the child during photography if necessary, but without influencing the child's choice of daily life situations. Before the interviews, the child will be asked to choose up to ten photos. Parents will be instructed not to influence the child's choice except if the images may be perceived as violating privacy.

Interviews will be conducted after the photography period. The child will be asked about the situation shown in the photo. For example, children will be asked about the context of the situation and why it was chosen, about objects and/or people shown, and how the child felt in and about the situation. The interviews will be conducted in the child's home or another environment chosen by the child. A parent can accompany the child if the child wishes, but will be instructed to remain silent throughout the interview.

PEI has been used to study children's perceptions and experiences in various contexts with different research goals (Einarsdottir, 2005; Epstein et al., 2006; Ford et al., 2017; Hong & Goh, 2019; McCloy et al., 2016). The approach is based on the idea of participatory research and emphasizes the "voice" of those being researched, questioning the acquisition and usefulness of knowledge, the power relationship between researchers and the researched, and the position of the "objective" researcher. Involving children as researchers is a valid and appropriate way to obtain information about the lives of children and adolescents (Kirby, 2001). PEI gives the participant control over contextual frames by determining which pictures to take and include for interviews. One of the advantages of using this method with children is that it can introduce content areas that might otherwise be overlooked or poorly understood by the researcher (Clark & Statham, 2005). The use of both visual and verbal components helps the child to express thoughts and possibly address sensitive aspects of family life (Zartler & Richter, 2014).

Timeline and Flowchart for the Project Components

- **Autumn 2020:** Completion of detailed project plan, ethics application, start recruiting participating municipalities, and finalizing study materials. Recruitment of doctoral student. Registration in Clinical Trials or equivalent.
- **Spring 2021:** Train data collectors such as PYC facilitators, social workers in study setup, recruitment and data collection. Start recruitment, data collection, and intervention.
- **Autumn 2021 – Spring 2023:** Continued recruitment, data collection, and intervention.
- **Autumn 2023 – Autumn 2024:** Compilation and interim reporting of results, scientific articles, and popular science communication.
- **Autumn 2024 – Autumn 2025:** Final reporting and publication of the doctoral thesis, compilation thesis.

See further flowchart (appendix 13) for how the different parts follow each other.

Study Implementation

The study will be conducted in collaboration with the social services units of several municipalities willing to contribute to the study and that already have trained PYC facilitators who can carry out the intervention. Participants will be recruited by social workers at the respective participating social services units.

Group Size, Recruitment, and Randomization

For the RCT study (Track I), 80 participants (n=40 per group) will be recruited. Of these, approximately 15 parents who have received PYC will be recruited for parent interviews. Children of parents allocated to PYC aged 3–5 years (n=20) and 5–6 years (n=12) will be asked to participate.

This project has two primary outcome measures, and group size analyses have been conducted for both goal achievement measured by COPM and responsiveness. The

group size calculation for COPM is based on a previous study of adults with cognitive impairment (early dementia, n=69, Mn 77.78 years) (Clare et al., 2010). To our knowledge, there are no randomized intervention studies where COPM has been used for individuals with IDD. COPM has two subscales: performance and satisfaction, and the analysis is based on findings from measurements on the performance scale (1-10). The intervention group and the control group had comparable values before the intervention, and after the intervention, the difference between the groups was 1.47 on the performance scale (SD 2.04). Group size calculation based on this data with 80% power and a significance level of p=0.05 shows that n=30 participants per group is sufficient (calculated using http://infovoice.se/fou).

Meta-analyses of responsiveness interventions have documented medium combined effects of d = .54 - .58 (Juffer et al., 2018; van IJzendoorn et al., 1996), with stronger effects for parents from risk groups who exhibited low responsiveness before the intervention. The present study includes parents at risk of neglect, who are at an increased risk of low responsiveness, and may therefore find stronger effects. At the same time, the meta-analyses have concerned interventions specifically focused on responsiveness, whereas PYC has a broader perspective on positive interaction, and it is therefore unclear to what extent they are applicable to PYC. Although a relatively recent Swedish study found reduced responsiveness in mothers with ID (Lindberg et al., 2017), there are no guiding intervention studies regarding responsiveness for parents with cognitive difficulties. Based on the meta-analytic average effects, n = 38 participants per group would be needed.

Overall, it is assessed that the study will need at least 60 participants; preliminary analyses can determine whether recruitment needs to continue until 80 participants are included. A randomized allocation sequence will be generated in collaboration with a biostatistician with a 1:1 allocation ratio. The allocation sequence will be concealed from recruiting social workers to avoid bias. For each recruited parent, the social worker will contact the research team, which will inform them of the assigned intervention (PYC or TAU). Additional blinding or masking is not possible in this study.

Research Team

Lena Almqvist, Associate Professor, Licensed Psychologist, Mälardalen University, lena.almqvist@mdh.se Maria Ayoub, Doctoral Student, Social Worker, Dalarna University, may@du.se Tommie Forslund, Researcher, Licensed Psychologist, Stockholm University, tommie.forslund@psychology.su.se Karin Fängström, Researcher, Licensed Psychologist, Uppsala Region, Academic Children's Hospital, Associate Researcher Uppsala University, karin.fangstrom@pubcare.uu.se Marie Holmefur, Professor, Licensed Occupational Therapist, Örebro University, marie.holmefur@oru.se Gunnel Janeslätt, Researcher, Licensed Occupational Therapist, Center for Clinical Research, Dalarna Region, gunnel.janeslatt@regiondalarna.se Karin Jöreskog, Director, Center for Disability Research, Uppsala University, karin.joreskog@cff.uu.se Eva Randell, Senior Lecturer, Social Worker, Dalarna University, era@du.se Thomas Strandberg, Professor, Social Worker, Dalarna University, tst@du.se Birgitta Wennberg, Researcher, Licensed

Occupational Therapist, Linköping University and Stockholm Region, Habilitation and Health, birgitta.wennberg@liu.se

Project Group

Agnetha Hammerin, MSc, Social Worker, Head of SUF Knowledge Center, agnetha.hammerin@regionuppsala.se Marie Holmberg, Head of Advice and Support, Social Services Enköping Municipality, marie.holmberg@enkoping.se Mats Jansson, Autism and Asperger Association, mats.jansson@autism.se Lydia Springer, Licensed Psychologist, Specialist/Support User Participation, SUF Knowledge Center, lydia.springer@regionuppsala.se Elisabeth Sundström Graversen, Attention, elisabeth.sundstrom-graversen@attention.se Judith Timoney, FUB, judith.timoney@fub.se Parents with IDD who have experiences with PYC will be included in the project group.

References

Adams, N. C., & Jarrold, C. (2012). Inhibition in autism: Children with autism have difficulty

inhibiting irrelevant distractors but not prepotent responses. *Journal of autism and developmental disorders*, *42*(6), 1052-1063.

Ainsworth, M. D. S., Bell, S. M., & Stayton, D. F. (1974). Infant-mother attachment and social development: Socialization as a product of reciprocal responsiveness to signals. In M. P. M. Richards (Ed.), *The integration of a child into a social world; the integration of a child into a social world* (pp. 99-135) Cambridge University Press, New York.

Alexius, K., & Hollander, A. (2014). Care assessments concerning involuntary removal of children from intellectually disabled parents. *J of Social Welfare and Family Law. no 3.* 295-310.

Almqvist, A.-L., & Almqvist, L. (2015). Making oneself heard–children's experiences of empowerment in Swedish preschools. *Early child development and care, 185*(4), 578-593.

Arvidsson, G., Buchholz, M., Forsmark, G., Hård, A., Jacobsson, C., Meden, D., . . .Janeslätt, G., Wennberg, B., (2013). Metoder för kognitivt stöd: Evidensbaserad habilitering, *EBH* (pp. 88). Stockholm: Föreningen Sveriges Habiliteringschefer http://www.habiliteringschefer.se/ebh/kognitivt/kognitivt.html#aktuella.

Azar, S. T., Stevenson, M. T., & Johnson, D. R. (2012). Intellectual disabilities and neglectful parenting: Preliminary findings on the role of cognition in parenting risk. *J of mental health research in intellectual disabilities, 5*(2), 94- 129.

Azar, S. T., Maggi, M. C., & Proctor, S. N. (2013). Practices changes in the child protection system to address the needs of parents with cognitive disabilities. *Journal of public child welfare, 7*(5), 610-632.

Bakermans-Kranenburg, M., van IJzendoorn, M. H., & Kroonenberg, P. M. (2004). Differences in attachment security between african-american and white children: Ethnicity or socio-economic status? *Infant Behavior & Development, 27*(3), 417-433.

Bernier, A., Carlson, S. M., Deschênes, M., & Matte-Gagné, C. (2012). Social factors in the development of early executive functioning: A closer look at the caregiving environment. *Developmental Science, 15*(1), 12-24.

Ben-Arieh, A. (2012). How do we measure and monitor the "state of our children"?: revisiting the topic in honor of Sheila B. Kamerman. *Children and Youth Services Review, 34*(3), 569-575.

Bokström, P., Fängström, K., Calam, R., Lucas, S., & Sarkadi, A. (2016). 'I felt a little bubbly in my tummy': Eliciting pre-schoolers' accounts of their health visit using a computer-assisted interview method. *Child: Care, Health and Development, 42*(1), 87-97.

Bøhren, I. E., & Stabrun, R. (2013). *«Fordi de ikke er i hop mer»: En kvalitativ studie av samtaleverktøyet In My Shoes i samtaler med barn i skilsmissekonflikter.* University of Oslo.

Booth, T., & Booth, W. (2004). Findings from a court study of care proceedings involving parents with intellectual disabilities. *J of Policy and Practice in Intellectual Disabilities, 1*(3-4), 179- 181.

Booth, T., & Booth, W. (2005). Parents with learning difficulties in the child protection system: experiences and perspectives. *J Intellect Disabil, 9*(2), 109- 129.

Booth, T., McConnel D., & Booth, W. (2006). Temporal Discrimination and Parents with Learning Difficulties in the Child Protection System. *British Journal of Social Work, 26*(6), 997-1015.

Bexkens, A., Ruzzano, L., Collot d'Escury-Koenigs, A. M. L., Van der Molen, M. W., & Huizenga, H. M. (2014). Inhibition deficits in individuals with intellectual disability: A meta-regression analysis. *Journal of Intellectual Disability Research*, *58*(1), 3-16.

Bruno, L. (2012). *Föräldrar med kognitiva svårigheter - att bryta ny mark*. Retrieved from

Brännström, L., Vinnerljung, B., Forsman, H., & Almquist, Y. B. (2017). Children placed in out-of-home care as midlife adults: Are they still disadvantaged or have they caught up with their peers? *Child Maltreatment, 22*(3), 205-214.

Calam, R., Cox, A., Glasgow, D., Jimmieson, P., & Groth Larsen, S. (2000). Assessment and therapy with children: Can computers help? *Clinical Child Psychology and Psychiatry, 5*(3), 329-343.

Calam, R., Jimmieson, P., Cox, A. D., Glasgow, D. V., & Groth Larsen, S. (2000). Can computer-based assessment help us understand children's pain? *European J of Anaesthesiology, 17*, 284-288.

Carswell, A., McColl, M. A., Baptiste, S., Law, M., Polatajko, H., & Pollock, N. (2004). The Canadian Occupational Performance Measure: a research and clinical literature review. *Can J Occup Ther, 71*(4), 210-222.

Charlton, L., Crank, M., Kansara, K., & Oliver, C. (1998). Still Screaming: Birth parents compulsorily separated from their children. *Manchester: After Adoption*.

Clare, L., Linden, D. E., Woods, R. T., Whitaker, R., Evans, S. J., Parkinson, C. H ..., Rugg, M. D. (2010). Goal-Oriented Cognitive Rehabilitation for People With Early-Stage

Alzheimer Disease: A Single-Blind Randomized Controlled Trial of Clinical Efficacy. *Am J Geriatr Psychiatry, 18*(10), 928-939.

Clark, A., & Statham, J. (2005). Listening to young children: Experts in their own lives. *Adoption & Fostering, 29*(1), 45-56.

Crittenden, P. M., & Bonvillian, J. D. (1984). The relationship between maternal risk status and maternal sensitivity. *American J of Orthopsychiatry, 54*(2), 250-262.

Cyr, C., Euser, E. M., Bakermans-Kranenburg, M., & Van Ijzendoorn, M. H. (2010). Attachment security and disorganization in maltreating and high-risk families: A series of meta-analyses. *Development and Psychopathology, 22*(1), 87-108.

Dahlberg, A., Fält, E., Ghaderi, A., Sarkadi, A., & Salari, R. (2019). Swedish norms for the Strengths and Difficulties Questionnaire for children 3–5 years rated by parents and preschool teachers. *Scandinavian J of Psychology*.

Dahlberg, A., Ghaderi, A., Sarkadi, A., & Salari, R. (2019). SDQ in the hands of fathers and preschool teachers—Psychometric properties in a non-clinical sample of 3–5-year-olds. *Child Psychiatry and Human Development, 50*(1), 132-141.

De Wolff, M., & van IJzendoorn, M. H. (1997). Sensitivity and attachment: A meta-analysis on parental antecedents of infant attachment. *Child Development, 68*(4), 571-591.

DiLillo, D., & Damashek, A. (2003). Parenting characteristics of women reporting a history of childhood sexual abuse. *Child Maltreatment*, *8*(4), 319-333.

Dowdney, L., & Skuse, D. (1993). Parenting provided by adults with mental retardation. *Child Psychology & Psychiatry & Allied Disciplines, 34*(1), 25-47.

Einarsdottir, J. (2005). Playschool in pictures: Children's photographs as a research method. *Early child development and care, 175*(6), 523-541.

Eklund, M., Bäckström, M., & Eakman, A. (2014). Psychometric properties and factor structure of the 13-item satsifaction with daily occupations scale when used with people with mental health problems. *Health Quality of Life Outcomes, 24*(12), 191. doi:10.1186/s12955-014-0191-3.

Elo, S., & Kyngäs, H. (2008). The qualitative content analysis process. *J of advanced nursing, 62*(1), 107-115.

Enemark Larsen, A., Rasmussen, B., & Christensen, J. R. (2018). Enhancing a Client-Centred Practice with the Canadian Occupational Performance Measure. *Occup Ther Int,*

Epstein, I., Stevens, B., McKeever, P., & Baruchel, S. (2006). Photo elicitation interview: Using photos to elicit children's perspectives. *International J of qualitative methods, 5*(3), 1-11.

Feldman, M. A. (1994). Parenting education for parents with intellectual disabilities: a review of outcome studies. *Res Dev Disabil, 15*(4), 299- 332.

Feldman, M. A., Ducharme, J. M., & Case, L. (1999). Using self-instructional pictorial manuals to teach child-care skills to mothers with intellectual disabilities. *Behavior Modification, 23*(3), 480- 497.

Fernqvist, S. (2015). Negotiating parenthood: Experiences of economic hardship among parents with cognitive difficulties. *J of Intellectual Disabilities, 19*(3), 215- 229.

Ford, K., Bray, L., Water, T., Dickinson, A., Arnott, J., & Carter, B. (2017). Auto-driven photo elicitation interviews in research with children: Ethical and practical considerations. *Comprehensive child and adolescent nursing, 40*(2), 111-125.

Fängström, K. (2017). *'I don't even remember anything': Optimising the choice of method when interviewing preschoolers.* (Ph.D. Ph.D.). Uppsala university, Uppsala.

Fängström, K., Bokström, P., Dahlberg, A., Calam, R., Lucas, S., & Sarkadi, A. (2016). In My Shoes – Validation of a computer assisted approach for interviewing children. *Child Abuse and Neglect, 58*, 160-172.

Fängström, K., Salari, R., Eriksson, M., & Sarkadi, A. (2017). The computer-assisted interview In My Shoes can benefit shy preschool children's communication. *PloS One, 12*(8).

George, C., & Solomon, J. (2008). The caregiving system: A behavioral systems approach to parenting. In J. Cassidy, & P. R. Shaver (Eds.), *2nd ed.; handbook of attachment: Theory, research, and clinical applications* pp. 833-856. The Guilford Press, New York.

Gillberg, C., & Geijer-Karlsson, M. (1983). Children born to mentally retarded women: A 1-21 year follow-up study of 41 cases. *Psychological Medicine, 13*(4), 891-894.

Gillespie, A., Best, C., & O'Neill, B. (2012). Cognitive function and assistive technology for cognition: a systematic review. *J Int Neuropsychol Soc, 18*(1), 1-19.

Gilmore, L., & Cuskelly, M. (2009). Factor structure of the parenting sense of competence scale using a normative sample. *Child: care, health and development, 35*(1), 48-55.

Glazemakers, I., & Deboutte, D. (2013). Modifying the 'Positive Parenting Program'for parents with intellectual disabilities. *J of Intellectual Disability Research, 57*(7), 616-626.

Goodman, R. (1997). The Strengths and Difficulties Questionnaire: a research note. *J of Child Psychology and Psychiatry, 38*(5), 581-586.

Goodman, R. (2001). Psychometric properties of the strengths and difficulties questionnaire. *J of the American Academy of Child and Adolescent Psychiatry, 40*(11), 1337-1345.

Graneheim, U. H., & Lundman, B. (2004). Qualitative content analysis in nursing research: concepts, procedures and measures to achieve trustworthiness. Nurse Education Today, 24(2), 105–112.

Granqvist, P., Forslund, T., Fransson, M., Springer, L., & Lindberg, L. (2014). Mothers with intellectual disability, their experiences of maltreatment, and their children's attachment representations: A small-group matched comparison study. *Attachment & human development, 16*(5), 417- 436.

Grasso, F., Atkinson, K., & Jimmieson, P. (2013). *In My Shoes - a computer assisted interview for communicating with children about emotions.* Paper presented at the 2013 Humaine Association Conference on Affective Computing and Intelligent Interaction (ACII), Geneva, Switzerland.

Harr, N., Dunn, L., & Price, P. (2011). Case study on effect of household task participation on home, community, and work opportunities for a youth with multiple disabilities. *Work, 39*(4), 445-453.

Mc Hugh, E., & Starke, M. (2015). Understanding support workers' competence development in working with parents with intellectual disability. *Journal of Intellectual Disabilities, 19*(4), 326-341.

Mc Hugh, E., & Starke, M. (2020). Investigating feasibility and fidelity of the Parenting Young Children program in Sweden. *80*, 101702.

McHugh, E., Wade, C., Mildon, R., & Starke, M. (2014). understanding professionals' competence development in working with parents with intellectual disability. *Journal of Applied Research in Intellectual Disabilities, 27*(4), 347.

Hershkowitz, I., Lamb, M. E., Orbach, Y., Katz, C., & Horowitz, D. (2012). The development of communicative and narrative skills among preschoolers: Lessons from forensic interviews about child abuse. *Child Development, 83*(2), 611-622.

Hindmarsh, G., Llewellyn, G., & Emerson, E. (2017). The Social-Emotional Well-Being of Children of Mothers with Intellectual Impairment: A Population-Based Analysis. *J of Applied Research in Intellectual Disabilities, 30*(3), 469-481.

Holmefur, M., Lidström-Holmqvist, K., Roshanay, A., Arvidsson, P., White, S., & Janeslätt, G. (2019). Pilot study of "Let's Get Organized" - a group intervention for improving time management *Americal Journal of Occupational Therapy, 73*(7305205020p1-7305205020p10). doi: doi:10.5014/ajot.2019.032631

Hong, R. T., & Goh, E. C. (2019). Using photo elicitation interviewing to access the subjective well-being of children from poor families within an affluent Asian society: Insights for service delivery. *Children and Youth Services Review, 96*, 430-438.

Höglund, B., & Larsson, M. (2013). Struggling for motherhood with an intellectual disability: A qualitative study of women's experiences in Sweden. *Midwifery, 29*(6), 698-704.

Höglund, B., Lindgren, P., & Larsson, M. (2012). Pregnancy and birth outcomes of women with intellectual disability in Sweden: a national register study. *Acta obstetricia et gynecologica Scandinavica, 91*(12), 1381-1387.

Ineland, J., Molin, M., & Sauer, L. (2009). *Utvecklingsstörning, samhälle och välfärd*: Gleerups Sweden.

Janeslätt G., & Hayat Roshanay, A. (2015). *Föräldraskap hos vuxna med ADHD eller Autismspektrumtillstånd, konsekvenser för barnet samt metoder för stöd. Systematisk kunskapsöversikt [Parents with ADHD or Autism consequences for the child and methods for support. A systematic review]* (2015:7).

Janeslätt, G., Jöreskog, K., Lindstedt, H., & Adolfsson, P. (2019a). Experiences of the maternal role and support in mothers with cognitive limitations who have children in placement. *Child & Family Social Work, 24*(4), 494-502.

Janeslätt, G., Larsson, M., Wickström, M., Springer, L., & Höglund, B. (2019b). An Intervention using the Tool-kit "Children – what does it involve?" and the Real-Care-Baby simulator among students with ID - a feasibility study. *Journal of Applied Research in Intellectual Disabilities*(32), 380-389. doi:10.1111/jar.12535

Juffer, F., Bakermans-Kranenburg, M. J., & van IJzendoorn, M. H. (2017). Pairing attachment theory and social learning theory in video-feedback intervention to promote positive parenting. *Current Opinion in Psychology, 15*, 189-194.

Kirby, P. (2001). Involving Young People in Research. In B. Franklin (Ed.), *Handbook of Children's Rights*. London: Routledge.

Kok, R., Thijssen, S., Bakermans-Kranenburg, M., Jaddoe, V. W. V., Verhulst, F. C., White, T., ...Tiemeier, H. (2015). Normal variation in early parental sensitivity predicts child structural brain development. *J of the American Academy of Child & Adolescent Psychiatry, 54*(10), 824-831.

Lange, A.M., Daley, D., Frydenberg, M., Houmann, T., Kristensen L.J., Rask C., ...Thomsen, P.H. (2018). Parent training for preschool ADHD in routine, specialist care: A Rrandomized controlled trial. *Journal of the American Academy of Child & Adolescent Psychiatry, 57*(8), 593-602.

Law, M., Baptiste, S., Carswell, A., McColl, M. A., Polatajko, H. J., & Pollock, N. (2005). *Canadian Occupational Performance Measure*. Ottawa, ON: CAOT Publications ACE.

Lord, C., Elsabbagh, M., Baird, G., & Veenstra-Vanderweele, J. (2018). Autism spectrum disorder. *The Lancet*, *392*(10146), 508-520.

Lindberg, L., Fransson, M., Forslund, T., Springer, L., & Granqvist, P. (2017). Maternal sensitivity in mothers with mild intellectual disabilities is related to experiences of maltreatment and predictive of child attachment: A matched-comparison study. *J of Applied Research in Intellectual Disabilities, 30*(3), 445-455.

Lindblad, I., Billstedt, E., Gillberg, C., & Fernell, E. (2013). An interview study of young adults born to mothers with mild intellectual disability. *J of Intellectual Disabilities*.

Llewellyn, G., McConnell, D., Honey, A., Mayes, R., & Russo, D. (2003a). Promoting health and home safety for children of parents with intellectual disability: a randomized controlled trial. *Research in Developmental Disabilities, 24*(6), 405-431.

Llewellyn, G., McConnell, D., & Ferronato, L. (2003b). Prevalence and outcomes for parents with disabilities and their children in an Australian court sample. *Child Abuse & Neglect, 27*(3), 235-251.

Malmberg, M., Rydell, A., & Smedje, H. (2003). Validity of the swedish version of the strengths and difficulties questionnaire (SDQ-swe). *Nordic J of Psychiatry,*

Mayes, R., & Llewellyn, G. (2009). What happens to parents with intellectual disability following removal of their child in child protection proceedings? *J Intellect Dev Disabil, 34*(1), 92- 95.

Mayes, R., Tozer, R., & Elder, M. (2011). An innovative support group for parents with intellectual disabilities whose children have been removed. *Developing Practice: The Child, Youth and Family Work Journal*(29), 58.

McCloy, L., White, S., Lee Bunting, K., & Forwell, S. (2016). Photo-Elicitation Interviewing to Capture Children's Perspectives on Family Routines. *J of Occupational Science, 23*(1), 82-95.

McColl, M. A., Law, M., Baptiste, S., Pollock, N., Carswell, A., & Polatajko, H. J. (2005). Targeted applications of the Canadian Occupational Performance Measure. *Can J Occup Ther, 72*(5), 298-300.

McConnell, D., Feldman, M., Aunos, M., & Prasad, N. (2011). Parental cognitive impairment and child maltreatment in Canada. *Child Abuse & Neglect, 35*(8), 621-632.

McConachie, H., Colver, A., Forsyth, R., Jarvis, S., & Parkinson, K. (2006). Participation of disabled children: how should it be characterised and measured? *Disability and Rehabilitation, 28*(18), 1157-1164.

McGaw, S., Ball, K., & Clark, A. (2002). The effect of group intervention on the relationships of parents with intellectual disabilities. *Journal of Applied Research in Intellectual Disabilities, 15*(4), 354-366.

McGaw, S., Shaw, T., & Beckley, K. (2007). Prevalence of psychopathology across a service population of parents with intellectual disabilities and their children. *J of Policy and Practice in Intellectual Disabilities, 4*(1), 11-22.

McHugh, E., Wade, C., Mildon, R., & Starke, M. (2014). Understanding professionals' competence development in working with parents with intellectual disability. *J of Applied Research in Intellectual Disabilities, 27*(4), 347.

McHugh, E., & Starke, M. (2015). Understanding support workers' competence development in working with parents with intellectual disability. *J of Intellectual Disabilities, 19*(4), 326-341.

McHugh, E. (2016). Implementing a program for parents with intellectual disability in Sweden: A feasibility study.

Mc Hugh, E., & Starke, M. (2020). Investigating feasibility and fidelity of the Parenting Young Children program in Sweden. *80, 101702.*

Mensas, L. (2014). *Parenting Young Children: PYC Rapport från tre års utvecklings- och implementeringsarbete*. Retrieved from FoU Sjuhärad Välfärd Högskolan i Borås 501 90 Borås www.fous.se: http://www.diva-portal.org/smash/person.jsf?pid=authority-person%3A43224&dswid=-81

Mildon, R., Wade, C., & Matthews, J. (2008). Considering the contextual fit of an intervention for families headed by parents with an intellectual disability: An exploratory study. *J of Applied Research in Intellectual Disabilities, 21*(4), 377-387.

Mineur, T., Bergh, S., & Tideman, M. (2009). Livssituationen för unga vuxna med lindrig utvecklingsstörning-en kunskapsöversikt baserad på skandinavisk forskning 1998-2009.

Nybom, J. (2005). Visibility and 'child view'in the assessment process of social work: cross-national comparisons. *International J of Social Welfare, 14*(4), 315-325.

Pecora, P. J., Sanders, D., Wilson, D., English, D., Puckett, A., & Rudlang-Perman, K. (2014). Addressing common forms of child maltreatment: evidence-informed interventions and gaps in current knowledge. *Child & Family Social Work, 19*(3), 321-332.

Reedtz, C., Handegård, B.H., & Mørch, W.T. (2011). Promoting positive parenting practice in primary pare: Outcomes and meachanisms of change in a randomized controlled risk reductional trial. *Scandinavian Journal of Psychology, 52,* 131-137.

Richardson JR, Peacock SJ, Hawthorne G, Iezzi A, Elsworth G, Day NA. Construction of the descriptive system for the Assessment of Quality of Life AQoL-6D utility instrument. *Health Qual Life Outcomes. 2012* Apr 17;10:38.

Rogers, H., & Matthews, J. (2004). The parenting sense of competence scale: Investigation of the factor structure, reliability, and validity for an Australian sample. *Australian Psychologist*, *39*(1), 88-96.

Roshanay, A. H., Janeslätt, G., White, S., & Lidström Holmqvist, K. (2019). Time Management Skills in Relation to General Self-Efficacy and Parental Sense of Competence in Individuals with and without Cognitive Disabilities *Cogent Psychology*. doi:10.1080/23311908.2019.1655981

Rosqvist, H. B., & Lövgren, V. (2013). Doing adulthood through parenthood: Notions of parenthood among people with cognitive disabilities. *ALTER-European Journal of Disability Research/Revue Européenne de Recherche sur le Handicap, 7*(1), 56-68.

Rydell, A., Berlin, L., & Bohlin, G. (2003). Emotionality, emotion regulation, and adaptation among 5- to 8-year-old children. *Emotion, 3*(1), 30-47.

Sakzewski, L., Boyd, R., & Ziviani, J. (2007). Clinimetric properties of participation measures for 5- to 13-year-old children with cerebral palsy: a systematic review. *Dev Med Child Neurol, 49*(3), 232-240.

SBU. (2018). Öppenvårdsinsatser för familjer där barn utsätts för våld och försummelse. Rapport 280. Statens beredning för medicinsk och social utvärdering.

Schalock, R. L., Bonham, G. S., Borthwick-Duffy, S. A., Bradley, V. J., Buntinx, W. H. E., Coulter, D. L., Craig, E. M., et al. (2010). *Intellectual disability: Definition, classification, and systems of supports*. Washington, DC: American Association on Intellectual and Developmental Disabilities.

Smedje, H., Broman, J., Hetta, J., & von Knorring, A. (1999). Psychometric properties of a Swedish version of the "Strengths and Difficulties Questionnaire.". *European Child and Adolescent Psychiatry, 8*(2), 63-70.

Sommer, D., Samuelsson, I. P., & Hundeide, K. (2009). *Child perspectives and children's perspectives in theory and practice* (Vol. 2). Dordrecht, Heidelberg: Springer.

Sonuga-Barke E.J.S., Daley, D., & Thompson, M. (2002). Does maternal ADHD reduce the effectiveness of parental trainging for prescool children's ADHD? *Journal of the American Academy of Child & Adolescent Psychiatry, 41*(6), 696-702.

Starke, M. (2005). *Föräldrar med utvecklingsstörning och deras barn - vad finns för kunskap?* (2005-123-3). Socialstyrelsen.

Starke, M. (2011). Supporting families with parents with intellectual disability: views and experiences of professionals in the field. *Journal of Policy and Practice in Intellectual Disabilities, 8*(3), 163-171.

Starke, M., Wade, C., Feldman, M. A., & Mildon, R. (2013). Parenting with disabilities Experiences from implementing a parenting support programme in Sweden. *J of Intellectual Disabilities, 17*(2), 145-156.

Stone, L. L., Janssens, J. M., Vermulst, A. A., Van Der Maten, M., Engels, R. C., & Otten, R. (2015). The Strengths and Difficulties Questionnaire: psychometric properties of the parent and teacher version in children aged 4-7. *BMC Psychol, 3*(1), 4.

Stovall-McClough, K., & Cloitre, M. (2006). Unresolved attachment, PTSD, and dissociation in women with childhood abuse histories. *J of Consulting and Clinical Psychology, 74*(2), 219-228.

Socialstyrelsen (2015). Effekter av föräldrastöd. Redovisning av en nationell utvärdering på uppdrag av Socialstyrelsen.

Thomson, J., & Thorpe, R. (2003). The importance of parents in the lives of children in the care system. *Children Australia, 28*(2), 25- 31.

Tøssebro, J., Midjo, T., Paulsen, V., & Berg, B. (2017). Prevalence, trends and custody among children of parents with intellectual disabilities in Norway. *J of Applied Research in Intellectual Disabilities, 30*(3), 533-542.

Thronsen, A. (2014). Normalization at any cost? A study of the parent education programme Parenting Young Children (PYC) as a model of preventive intervention in Norway In S. A. Crabtree (Ed.), *Diversity and the Process of Marginalisation and Otherness: giving voice to hidden themes – a European perspective*. London: Whiting &Birch

Thronsen, A., & Young, E. . (2015). *PYC i Norge. Foreldreveiledning til foreldre med kognitive funksjonsnedsettelser i møte med barnevernet 2014-2016. En rapport om erfaringer ved oversettelse av foreldreveiledningsprogrammet Parenting Young*

Children (PYC) til norsk språk og kultur. Retrieved from Porsgrunn: http://hdl.handle.net/11250/2439147

van IJzendoorn, M. H., Juffer, F., & Duyvesteyn, M. G. C. (1996). Breaking the intergenerational cycle of insecure attachment: A review of the effects of attachment-

based interventions on maternal sensitivity and infant security. *Annual Progress in Child Psychiatry & Child Development.* 157-183.

Wade, C., Llewellyn, G., & Matthews, J. (2008). Review of parent training interventions for parents with intellectual disability. *J of Applied Research in Intellectual Disabilities, 21*(4), 351- 366.

Wahn, E. H., & Nissen, E. (2008). Sociodemographic background, lifestyle and psychosocial conditions of Swedish teenage mothers and their perception of health and social support during pregnancy and childbirth. *Scandinavian J of Social Medicine, 36*(4), 415-423.

Weiber, I., Berglund, J., Tengland, P. A., & Eklund, M. (2011). Children born to women with intellectual disabilities - 5-year incidence in a Swedish county. *J Intellect Disabil Res, 55*(11), 1078-1085. doi:10.1111/j.1365-2788.2011.01441.x

Willcutt, E. G., Doyle, A. E., Nigg, J. T., Faraone, S. V., & Pennington, B. F. (2005). Validity of the executive function theory of attention-Deficit/Hyperactivity disorder: A meta-analytic review. Biological Psychiatry, 57(11), 1336-1346.

Willcutt, E. G., Nigg, J. T., Pennington, B. F., Solanto, M. V., Rohde, L. A., Tannock, R., ... & Lahey, B. B. (2012). Validity of DSM-IV attention deficit/hyperactivity disorder symptom dimensions and subtypes. *Journal of abnormal psychology*, *121*(4), 991.

Wressle, E., Eeg-Olofsson, A. M., Marcusson, J., & Henriksson, C. (2002). Improved client participation in the rehabilitation process using a client-centred goal formulation structure. *Journal of rehabilitation medicine*, *34*(1), 5-11.

Zartler, U., & Richter, R. (2014). My family through the lens. Photo interviews with children and sensitive aspects of family life. *Children & Society, 28,* 42-54
